# Longitudinal MR-based proton-density fat fraction (PDFF) and T2* for the assessment of associations between bone marrow changes and myelotoxic chemotherapy

**DOI:** 10.1007/s00330-023-10189-y

**Published:** 2023-09-11

**Authors:** Felix G. Gassert, Julia Kranz, Florian T. Gassert, Benedikt J. Schwaiger, Christian Bogner, Marcus R. Makowski, Leander Glanz, Jonathan Stelter, Thomas Baum, Rickmer Braren, Dimitrios C. Karampinos, Alexandra S. Gersing

**Affiliations:** 1 Department of Radiology, Klinikum Rechts der Isar, School of Medicine, Technical University of Munich, Ismaninger Strasse 22, 81675 Munich, Germany; 2 Department of Neuroradiology, Klinikum Rechts der Isar, School of Medicine, Technical University of Munich, Munich, Germany; 3 Department of Oncology, Klinikum Rechts der Isar, School of Medicine, Technical University of Munich, Munich, Germany; 4 Department of Neuroradiology, University Hospital of Munich, Ludwig-Maximilians University Munich, Munich, Germany

**Keywords:** Magnetic resonance imaging, Drug therapy, Bone density, Spine

## Abstract

**Objectives:**

MR imaging-based proton density fat fraction (PDFF) and T2* imaging has shown to be useful for the evaluation of degenerative changes in the spine. Therefore, the aim of this study was to investigate the influence of myelotoxic chemotherapy on the PDFF and T2* of the thoracolumbar spine in comparison to changes in bone mineral density (BMD).

**Methods:**

In this study, 19 patients were included who had received myelotoxic chemotherapy (MC) and had received a MR imaging scan of the thoracolumbar vertebrates before and after the MC. Every patient was matched for age, sex, and time between the MRI scans to two controls without MC. All patients underwent 3-T MR imaging including the thoracolumbar spine comprising chemical shift encoding-based water-fat imaging to extract PDFF and T2* maps. Moreover, trabecular BMD values were determined before and after chemotherapy. Longitudinal changes in PDFF and T2* were evaluated and compared to changes in BMD.

**Results:**

Absolute mean differences of PDFF values between scans before and after MC were at 8.7% (*p *= 0.01) and at −0.5% (*p *= 0.57) in the control group, resulting in significantly higher changes in PDFF in patients with MC (*p *= 0.008). BMD and T2* values neither showed significant changes in patients with nor in those without myelotoxic chemotherapy (*p *= 0.15 and *p *= 0.47). There was an inverse, yet non-significant correlation between changes in PDFF and BMD found in patients with myelotoxic chemotherapy (*r *= −0.41, *p *= 0.12).

**Conclusion:**

Therefore, PDFF could be a useful non-invasive biomarker in order to detect changes in the bone marrow in patients receiving myelotoxic therapy.

**Clinical relevance statement:**

Using PDFF as a non-invasive biomarker for early bone marrow changes in oncologic patients undergoing myelotoxic treatment may help enable more targeted countermeasures at commencing states of bone marrow degradation and reduce risks of possible fragility fractures.

**Key Points:**

- Quantifying changes in bone marrow fat fraction, as well as T2* caused by myelotoxic pharmaceuticals using proton density fat fraction, is feasible.
- *Proton density fat fraction could potentially be established as a non-invasive biomarker for early bone marrow changes in oncologic patients undergoing myelotoxic treatment.*

## PACSPicture archiving and communication system

PDFFProton-density fat fraction

ROIRegion of interest

## Introduction

Myelotoxic chemotherapy regimen with bone marrow toxicity is one of the limiting factors in the treatment of cancer patients [1, 2]. The bone marrow consists of red marrow including leukocytes, thrombocytes, and hematopoietic stem cells, and yellow marrow, which reflects a reticular network primarily filled with lipids. Yellow marrow therefore reflects a higher fat content. While the hematopoietic red bone marrow is mainly located in the flat bones, vertebrae, sternum, and pelvis, the fatty yellow bone marrow can primarily be found in the cavities of the long bones. Myelotoxic chemotherapy leads to the reduction of hematopoietic cells and the hematopoietic function in the red marrow and also enhanced the differentiation of stem cells towards adipocytes, resulting in higher fat fractions of the yellow marrow [3]. These changes result in reduced hematopoiesis, loss of bone minerals, and a higher risk of fractures [3, 4]. Early detection of bone marrow conversion is crucial to avoid osteoporosis by early induction of therapies to reduce risks of possible fragility fractures [5].

Currently, dual-energy X-ray absorptiometry (DXA) and dedicated quantitative CT are the clinical standards for the evaluation of the effects of myelotoxicity through imaging [6]. Nevertheless, these techniques require additional radiation exposure and lead to extra costs. Previous studies have shown a significant gap in osteoporosis treatment, in particular in the elderly [7]. Especially in patients with an underlying oncologic disease, it is crucial to close this gap and avoid fragility fractures through bone anti-resorptive therapy. Previous studies have shown, that due to the shift of differentiation of mesenchymal stem cells from osteoblasts to adipocytes, osteoporosis is associated with an increase in bone marrow fat fraction (BMFF) [8, 9]. MRI-based proton density fat fraction (PDFF) is a reliable tool for the assessment of vertebral bone marrow water-fat composition allowing for quantitative assessment of bone marrow fat through PDFF maps without the downside of radiation exposure [10–12]. Further studies outlined a negative correlation between the BMFF and the BMD, measured through attenuation in CT scans [13–15].

A further previous study showed that water-fat MR imaging is sensitive to changes in red and yellow marrow composition and therefore postulates that this technique can be used for qualitative and quantitative assessment of treatment-induced marrow damage [16]. While the mentioned study focused on gynecologic cancer patients and on level L4 only, a different previous study additionally assessed the magnitude, rate, and pattern of change in several vertebrae of patients receiving highly myelotoxic pelvic chemotherapy and radiotherapy compared to patients receiving radiotherapy alone [17]. Further studies showed that T2* measurements of the vertebra correlated with the BMD, also suggesting decreased susceptibility due to remodeled and decreased trabecular bone [18, 19]. Nevertheless, so far there has been no study simultaneously assessing bone marrow changes through both PDFF and T2* in a heterogeneous patient group receiving myelotoxic chemotherapy compared to a control group receiving non-myelotoxic chemotherapy.

Therefore, the goal of this study was to evaluate the influence of myelotoxic chemotherapy on PDFF and T2* changes and compare these changes to the current standard of care imaging for osteoporosis. This could help provide a non-invasive biomarker for bone marrow changes and fill the diagnostic gap in osteoporosis treatment of oncologic patients.

## Methods

### Patient selection and study design

In this study, we retrospectively included patients who received myelotoxic chemotherapy and underwent an MR examination in our institution from March 2018 until April 2021 as part of the clinical routine diagnostic workup. Only patients who received MR-Scans within 2 months before and at least two months after chemotherapy were used for further analysis. Additionally, if available CT-Scans of those patients before and after chemotherapy were analysed from which a quantitative trabecular BMD measurement could be derived [20, 21].

Myelotoxic chemotherapeutics were confirmed by an expert in oncology (18 years of experience) and included FOLFIRINOX (*n *= 8), Sorafenib (*n *= 5), Gemcitabine (*n *= 4), and Gemcitabine + Cisplatin (*n *= 2). Patients who have already received myelotoxic chemotherapy before the first MR scan and patients who also received radiotherapy of the abdomen were excluded from the analysis.

Overall, 19 consecutive patients were included in this study (mean age 69 ± 7 years). Each of these patients was matched with two control patients regarding sex, age (± 5 years), duration of chemotherapy (± 3 years), and time between MRI scans (± 180 days).

### Magnetic resonance imaging and PDFF assessment

PDFF and T2* measurements were extracted using a chemical shift encoding-based water-fat separation technique in a 3 T MR imaging system (Ingenia, Philips Healthcare, Release 5.4). A six-echo 3D multi-echo gradient-echo sequence was used to acquire all echoes in a single TR, using bipolar gradients. To reduce the scan time a Compressed SENSE factor of 4 was employed. The combination of CS and SENSE, as well as the reconstruction, were based on the vendor’s implementation (Compressed SENSE, Philips Healthcare). The six echoes were acquired with an axial acquisition, using the following parameters: repetition time TR/ first echo time TE1/ echo time step ΔTE = 7.8/1.35/1.1 ms, field of view (FOV) = 300 × 400 × 150 mm^3^, acquisition voxel size = 2 × 3 × 6 mm^3^, reconstruction voxel size = 1.13 × 1.13 × 6 mm^3^, receiver bandwidth = 1678 Hz/pixel, frequency direction = anterior/posterior (A/P), 1 average, scan time = 9.3 s, using a combination of a 16-channel torso coil array and an inbuilt table posterior 12-channel coil array. To minimize T1-bias effects a flip angle of 3° was used. Complex multi-echo gradient-echo images were provided as input to the fat quantification routine provided by the vendor (mDixon Quant, Philips Healthcare), accounting for the multi-peak fat spectrum based on the fat spectrum from [22] and considering a single T2* decay. The resulting PDFF map represents the ratio of the fat signal over the sum of fat and water signals. PDFF and T2* maps were extracted.

Slices included at least two lumbar vertebrae. Segmentations of the vertebral bodies were manually performed on the PDFF and T2* maps according to previous literature [23, 24] using the Picture archiving and communication system (PACS) workstations. Cortical bone was not included in the analysis. Vertebrae with fractures or degenerative changes, e.g., Modic changes, were excluded from segmentation. The mean PDFF and T2* values were calculated from three slices for each vertebra and in a second step, the mean PDFF and T2* for each patient were calculated as the mean averaged over the respective spines of each patient.

The intra-reader reproducibility was calculated by segmenting the ROIs of a random sample of five subjects a second time after eight weeks and reanalyzing the measurements.

### Computed tomography and trabecular BMD measurements

Due to retrospective analysis, CT images were acquired on different CT systems. CT scans before and after therapy were available in 17 of 19 patients receiving myelotoxic chemotherapy and in 34 of 38 patients of the control group with a range of 1 to 39 days between the CT and respective MRI scans. Trabecular BMD values were derived from asynchronously calibrated quantitative CT examinations in 3 mm reformations. Hounsfield unit (HU) values were measured for at least two lumbar vertebrae in three slices in the axial plane using the IDS7 PACS (Sectra AB). Vertebrae with fractures or degenerative changes, e.g., Modic changes, were excluded from segmentation. Trabecular BMD values were then calculated from the average HU values with respective conversion equations, which had been developed previously with phantom calibration [25].

### Statistical analysis

All statistical analyses were performed using the statistical package R version 3.2.4 (R Foundation for Statistical Computing). Absolute differences in PDFF, T2*, and BMD values before and after chemotherapy were calculated. Also, in order to set the changes of these values into a context with accordingly baseline values, changes of these values between time points relative to the baseline values were calculated. All statistical tests were performed two-sided with a level of significance (α) of 0.05. Pearson’s correlation coefficient was used for the evaluation of the correlation between changes in PDFF and BMD. Intra-rater reproducibility for PDFF, T2*, and trabecular BMD values was assessed by calculating the intraclass correlation coefficient (ICC).

## Results

In this study, we included 57 patients in total (9 female, 67.8 ± 7.4 years). Nineteen patients received myelotoxic chemotherapy and were frequency matched for sex, age, and time difference between scans to 38 control subjects. The distribution of the patient characteristics between groups can be found in Table 1.

**Table 1:** Characteristics of patients with and without myelotoxic chemotherapy

	myel. CTx (*n* = 19)	Controls (*n* = 38)	All (*n* = 57)	*p* value
Sex				
Women	3	6	9	1
Men	16	32	48	
Age [years]	68.5 ± 6.6	67.4 ± 7.8	67.8 ± 7.4	0.569
Time between scans [months]	9.1 ± 3.7	7.9 ± 4.1	8.3 ± 4.0	0.265

*Myel.* myelotoxic; *CTx* chemotherapy

The time between the first (before myelotoxic chemotherapy) and the second MRI scan (after myelotoxic chemotherapy) was at 293.6 ± 114.5 days for the group which received myelotoxic chemotherapy and at 253.1 ± 127.9 days for the control group, with no significant difference between groups (*p *= 0.13). The time between the beginning of the myelotoxic chemotherapy and the second MRI scan was at 172.5 ± 107.2 days.

**Fig. 1:**
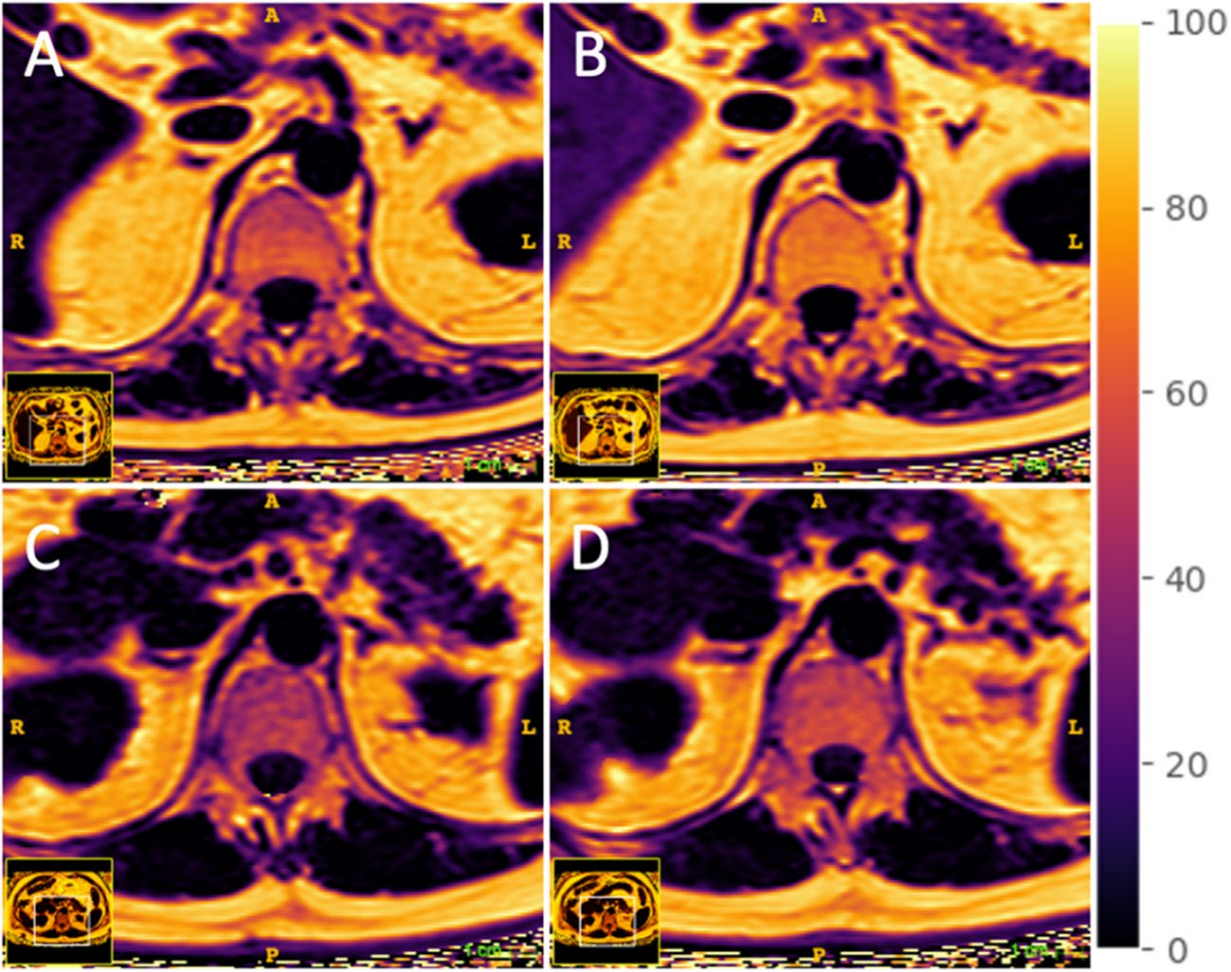
Axial slice of the lumbar spine showing the proton-density fat fraction of a patient before (**A**) and 6 months after (**B**) myelotoxic chemotherapy as well as patient without myelotoxic chemotherapy at baseline (**C**) and at 6-month follow-up (**D**)

Figure 1 shows images of a PDFF in a patient with and without myelotoxic chemotherapy. Absolute differences between scans before and after myelotoxic chemotherapy were evaluated, resulting in a significant increase in PDFF values with a mean difference of 8.6% (IQR: 0.6% to 11.2%, *p *= 0.01). The control group showed a mean difference of -0.5% (IQR: −2.6 to 3.5%, *p *= 0.57) of the PDFF values with significantly higher changes in patients with myelotoxic chemotherapy (Mean difference=9.1%; *p *= 0.008).

Relative differences in PDFF values before and after myelotoxic chemotherapy were at 25.7% (IQR: 1.1 to 29.6 %), whereas the control group showed relative differences of −0.7 % (IQR: −6.2 to 6.9 %) also resulting in significantly higher changes in patients with myelotoxic chemotherapy (Mean difference = 26.4%; *p *= 0.012).

Furthermore, absolute and relative differences in T2* values were evaluated for patients with and without myelotoxic chemotherapy. For T2* values mean differences were at 1.0 (IQR: −0.1 to 2.8, *p *= 0.27) for patients with myelotoxic chemotherapy and at 0.6 (IQR: −1.2 to 2.1, *p *= 0.84) for patients without myelotoxic chemotherapy. Relative differences of T2* values before and after myelotoxic chemotherapy were at 13.8 % (IQR: −1.3 to 33.8 %), whereas the control group showed relative differences of 4.4 % (IQR: −5.6 to 9.9 %).

There was no significant difference between groups for both, absolute and relative T2* value changes (*p *= 0.41/*p *= 0.31).

**Fig. 2:**
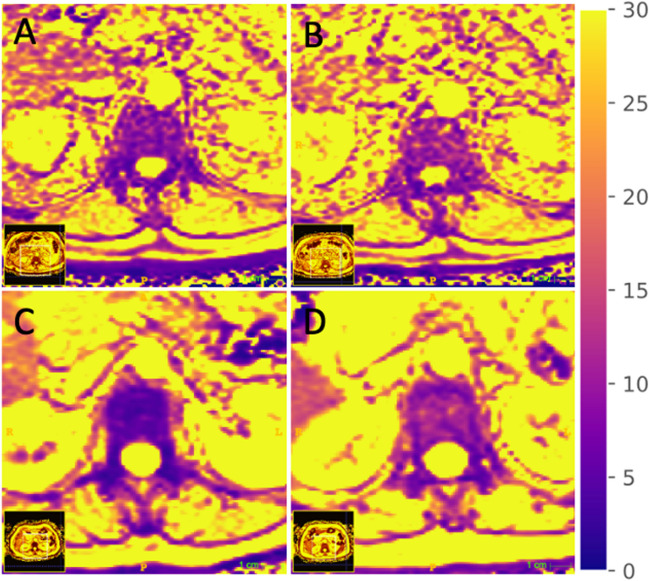
Axial slice of the lumbar spine showing T2* maps (in ms) of a patient before (**A**) and 6 months after (**B**) myelotoxic chemotherapy as well as a patient without myelotoxic chemotherapy at baseline (**C**) and at 6-month follow-up (**D**)

Additionally, based on the CT data, absolute and relative differences in BMD were calculated for both groups with and without chemotherapy. Mean differences of BMD were at −14.9 (IQR: −47.5 to −3.5, *p *= 0.15) for patients with myelotoxic chemotherapy and at −6.4 (IQR: −24.7 to 6.2, *p *= 0.47) for patients without myelotoxic chemotherapy, resulting in relative changes of BMD of −8.3% (IQR: −39.5 to 6.5%) for patients with myelotoxic chemotherapy and of −5.1% (IQR: −22.9 to 5.3%) for patients without myelotoxic chemotherapy. There was no significant difference in absolute and relative changes between groups (*p *= 0.45/*p *= 0.78). Changes for all three, PDFF, T2*, and BMD values in patients with and without myelotoxic chemotherapy are shown in Fig. 2. The intrareader agreement measurements were excellent for PDFF (ICC 0.98 [95% CI, 0.96–0.99]), T2* (ICC 0.97 [95% CI, 0.95–0.99]), and trabecular BMD values (ICC 0.99 [95% CI, 0.98–0.99]).

**Fig. 3:**
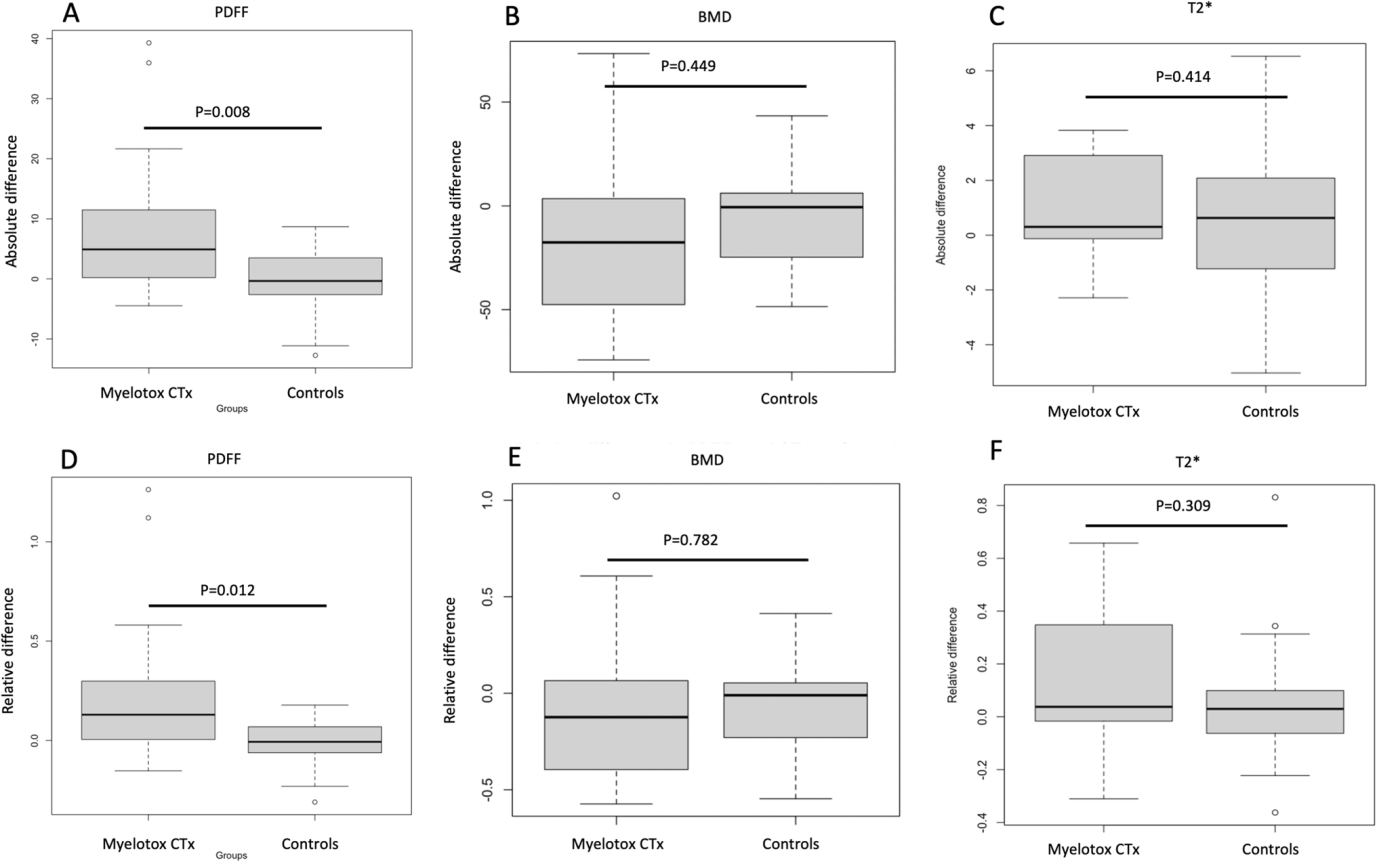
Absolute (**A**, **B**, **C**) and relative (**D**, **E**, **F**) differences in PDFF (**A**, **D**) and BMD (**B**, **E**) and T2* (**C**, **F**) between patients with and without myelotoxic chemotherapy. Myelotox = myelotoxic; CTx = chemotherapy; PDFF = Proton density fat fraction; BMD = Bone mineral density

**Fig. 4:**
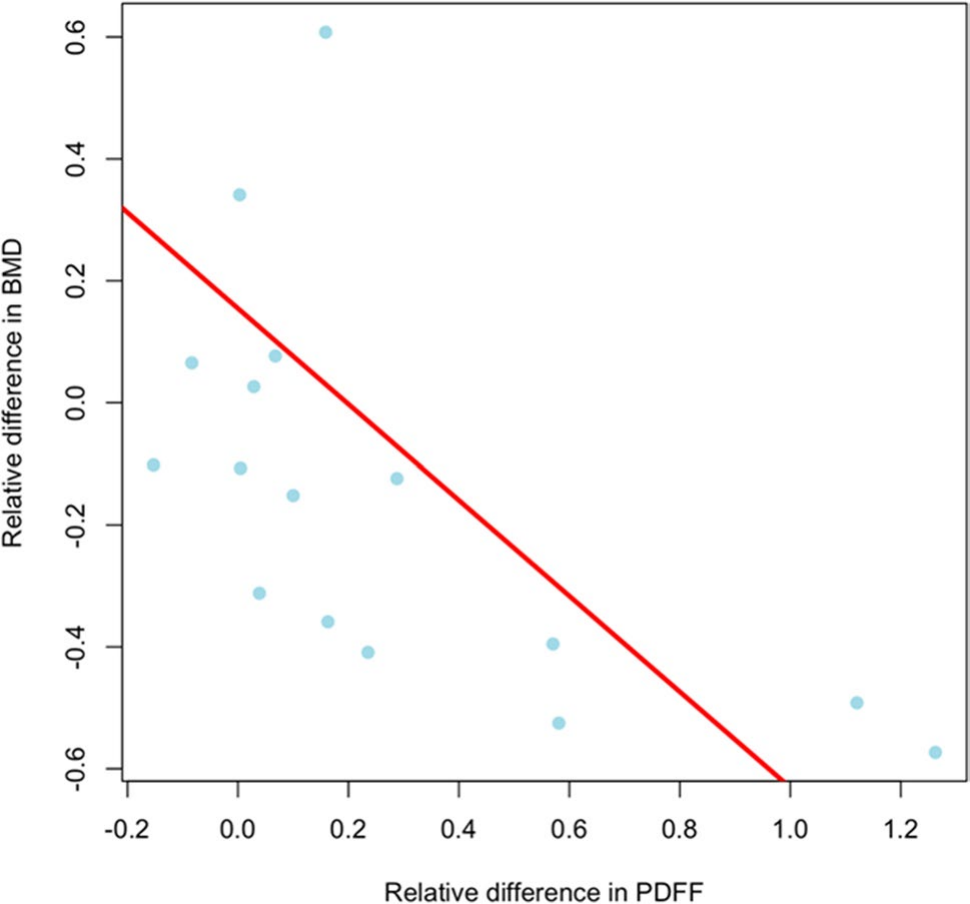
Scatterplot of the correlation of relative differences in PDFF and BMD in patients with myelotoxic chemotherapy. PDFF = Proton density fat fraction; BMD = Bone mineral density

The correlation between BMD and PDFF was evaluated for changes during myelotoxic chemotherapy and was *r* = −0.405 showing an inverse correlation between changes in BMD and PDFF in patients during myelotoxic chemotherapy (*p *= 0.12) (Fig. 3). The correlation between BMD and PDFF was evaluated for changes during myelotoxic chemotherapy and was *r* = −0.41 showing a negative correlation between changes in BMD and PDFF in patients during myelotoxic chemotherapy (*p *= 0.12) as shown in Fig. 4. The correlation coefficient between changes in BMD and PDFF in the control group was *r* = −0.24 (*p *= 0.23).

## Discussion

In this study, we evaluated the influence of myelotoxic chemotherapy on changes in MRI-based proton density fat fraction (PDFF) and T2* longitudinally and compared these changes to the changes in bone mineral density (BMD) values derived from quantitative computed tomography. Results showed, that absolute mean differences in PDFF values between scans before and after myelotoxic chemotherapy were significantly higher in patients receiving myelotoxic chemotherapy as compared to the control group. BMD and T2* values on the other hand neither showed significant changes in patients with nor in those without myelotoxic chemotherapy.

PDFF has shown to be a reliable tool for the assessment of vertebral bone marrow water-fat composition allowing for radiation-free quantitative assessment of bone marrow fat through PDFF maps [10–12]. It is derived from chemical shift encoding-based water-fat separation and measures a map of the density of hydrogen protons attributable to fat, normalized by the total hydrogen proton density from all mobile proton species, which enables fairly precise estimation of the fat volume fraction as a result of the almost equal relative proton densities of fat and water [26, 27].

Several clinical studies were performed on evaluating osteoporosis and bone marrow changes through PDFF and T2* maps in specific patient groups [15, 16, 18, 19]. Gassert et al showed that PDFF measures of the vertebral bone marrow can differentiate between patients with and without osteoporotic vertebral fractures [15], whereas Leonhardt et al propose T2* mapping of vertebral bone marrow to allow for assessing osteoporosis as well as the trabecular microstructure and enables a radiation-free differentiation between patients with low-energy osteoporotic and high-energy traumatic vertebral fractures [19]. Nevertheless, to our knowledge until today, no study has assessed bone marrow changes using both, PDFF as well as T2* measurements in the patient group receiving myelotoxic chemotherapy compared to a control group without myelotoxic chemotherapy.

In our study, we observed a weak negative correlation between PDFF and BMD values in patients who received myelotoxic chemotherapy. Although these results were non-significant, they are in line with previous literature. A study by Kühn et al outlined an inverse, moderate correlation between PDFF and BMD measured through DXA in a collective of 51 patients [13]. A further previous study analysed data from 400 healthy individuals, the BMD measurements assessed for differences in bone marrow composition and investigated the relationship between BMD and PDFF. Even after correcting for differences in bone marrow adipose composition and age in this study also an inverse correlation between BMD and PDFF values was found [28]. However, this previous study was performed using a cross-sectional design only.

Performing a longitudinal analysis of images of patients who received myelotoxic chemotherapy, we observed a significantly higher increase in PDFF compared to an untreated control group for both, absolute and relative changes, showing the decrease of the fat fraction in the spine as an effect of the myelotoxicity of the chemotherapy. These results are in line with a study by Carmona et al showing a correlation of PDFF with the intensity of radiochemotherapy in 19 patients [17]. While this study only included patients who received myelotoxic chemotherapy and no control group, we were able to prove this effect in a case-control design including both patients with and without myelotoxic chemotherapy. In addition to that effect, we observed that changes in PDFF were significant, whereas BMD did not show any significant changes as compared to the control group within the study period. This indicates that PDFF measurements may detect accelerated degeneration processes of the spine based on changes in the bone marrow at earlier stages than bone matrix-based BMD measurements and may serve as a potential sensitive tool to detect myelotoxic effects. Yet, future studies with larger study populations are needed in order to assess this potential further. T2* mapping -just like BMD- is capable of tracking bone matrix changes. Comparable to the BMD, the T2* values in our study also did not show any significant changes over time in patients treated with myelotoxic chemotherapy. These results are in line with a previous study by Wu et al, who showed a correlation between BMD and T2* values [18].

Compared to BMD and T2*, which evaluate bone matrix changes, PDFF focuses on changes in bone marrow fat content. Previous studies showed that fat fraction measurements through MRI are a reliable tool for the assessment of vertebral bone marrow water-fat composition, allowing for quantitative assessment of bone marrow fat through PDFF maps and that changes in PDFF correlate to peripheral blood cell counts [10–12, 14, 17]. Myelotoxic chemotherapy reduces hematopoietic cells in the red marrow, enhances differentiation of stem cells towards adipocytes, results in higher fat fractions of the yellow marrow, and leads to loss of bone minerals and a higher risk of fractures [3, 4]. PDFF could serve as a parameter of bone marrow conversion and therefore enable more targeted countermeasures at commencing states of bone marrow degradation to reduce risks of possible fragility fractures.

Nevertheless, this study has some limitations. Firstly, the T2* maps are relatively noisy, due to a SENSE factor of 4. Future studies in larger study cohorts need to be performed using R2* maps. Secondly, the number of patients included in this study is still relatively low which might lead to a lack of significance for BMD changes simply as a result of a low number of patients included. Besides larger patient numbers, future studies should include both, longer time frames but also measurements of PDFF and BMD values at multiple time points in order to show the relation and changes of these values over time and prove the potentially higher sensitivity of PDFF to bone structure changes in its early phases.

To conclude, this study is the first to show the feasibility of quantifying changes in bone and bone marrow fat fraction caused by myelotoxic chemotherapy in a longitudinal setting using MR-based PDFF and T2* measurements compared to patients who did not receive myelotoxic pharmaceuticals. Therefore, PDFF could potentially be useful as an early tool to detect changes in the bone marrow in patients receiving myelotoxic therapy and enable early countermeasures. Consequently, the results of this study could help establish a non-invasive biomarker for bone marrow changes in oncologic patients undergoing myelotoxic treatment.

## Abbreviations

BMD: Bone mineral density
BMFF: Bone marrow fat fraction
CT: Computed tomography
CTx: Chemotherapy
DXA: Dual-energy X-ray absorptiometry
FOV: Field of view
ICC: Intraclass correlation coefficient
MC: Myelotoxic chemotherapy
MR: Magnetic resonance
Myel.: Myelotoxic

## Declarations

### Guarantor

The scientific guarantor of this publication is Alexandra Gersing.

### Statistics and biometry

No complex statistical methods were necessary for this paper.

### Methodology

- Retrospective
- case-control study
- performed at one institution
